# Self-care strategies and sources of knowledge on menstruation in 12,526 young women with dysmenorrhea: A systematic review and meta-analysis

**DOI:** 10.1371/journal.pone.0220103

**Published:** 2019-07-24

**Authors:** Mike Armour, Kelly Parry, Mahmoud A. Al-Dabbas, Christina Curry, Kathryn Holmes, Freya MacMillan, Tania Ferfolja, Caroline A. Smith

**Affiliations:** 1 NICM Health Research Institute, Western Sydney University, Sydney, Australia; 2 Translational Health Research Institute, Western Sydney University, Sydney, Australia; 3 Centre for Educational Research, Western Sydney University, Sydney, Australia; 4 School of Science and Health, Western Sydney University, Sydney, Australia; Swinburne University of Technology, AUSTRALIA

## Abstract

Introduction: Dysmenorrhea (period pain) is common and affects around three quarters of all young women under the age of 25. The majority of young women, for a variety of reasons, think of period pain as ‘normal’ and something to be managed or endured. This normalisation of pain often is reinforced by family and friends and results in young women using self-care strategies to manage their pain rather than seeking medical advice. This systematic review and meta-analysis examined observational studies reporting on the prevalence of different types of self-care, both pharmaceutical and non-pharmaceutical, self-rated effectiveness of self-care and the sources of information on menstruation in young women under 25 Methods: A search of Medline, PsychINFO, EMBASE and CINAHL in English was carried out from 1980 to December 2018. Studies that reported on menstrual self-care strategies in young women were included. Results: Nine hundred and forty-seven articles were screened. Twenty-four studies including 12,526 young women were eligible and included in the meta-analysis. Fifteen studies were from low, lower-middle or upper-middle-income countries (LMIC) and nine studies were from high income countries (HIC). Self-care was used by over half of all young women (55%, 95%CI 34.1–74.3) with both pharmaceutical (48%, 95%CI 40.0–57.0) and non-pharmaceutical (51.8%, 95%CI 31.3–71.7) options used. Paracetamol was the most common analgesic used (28.7%, 95%CI 19.6–39.9) but did not always provide sufficient pain relief in almost half of those using it. Contraceptive use was significantly higher (P<0.001) in HIC (22%) compared to LMIC (1%). Only 11% (95%CI 8.4–15.2) of young women reported seeing a medical doctor for their period pain. Conclusions: Self-care usage, both pharmaceutical and non-pharmaceutical, was common, but young women were not necessarily choosing the most effective options for pain management. High-quality information on self-care for period pain is urgently needed.

## Introduction

Dysmenorrhea (period pain) is common and our recent meta-analysis shows dysmenorrhea affects around three quarters of all young women under the age of 25 worldwide[1]. Primary dysmenorrhea is defined as menstrual pain in the absence of underlying pathology [2, 3] and is the most common cause of dysmenorrhea in young women under the age of 25 [4]. In addition to painful cramps, many women with dysmenorrhea experience other menstrual related symptoms including back and thigh pain, headaches, diarrhoea, nausea and vomiting [5].

Dysmenorrhea or its associated symptoms often result in a reduction in classroom performance and increased absenteeism at school and tertiary education[1]. Despite this negative impact, most young women frame period pain as a normal part of being a woman[6], a common theme across varying geographic and ethnic boundaries[7, 8]. In addition, some countries such as Sri Lanka, Nigeria and India, have traditionally held strong taboos related to menstruation [9–11]. This in turn may impact how women manage their menstrual pain due to their beliefs and attitudes and/or a lack of accurate information[12].

In managing their period pain, many young women primarily use self-care. Self-care includes physical (e.g exercise, stretching or rest), pharmacological (e.g analgesic medication), non-pharmacological (e.g herbal medicine, heat) or psychological strategies (e.g prayer or meditation) that are usually undertaken by women themselves without seeking medical advice [13]. In the case of period pain these commonly include over the counter (OTC) analgesic medications (e.g. ibuprofen and paracetamol), rest and the application of heat [7, 8, 14–22]. Lack of satisfactory pain relief and effective medical interventions in primary dysmenorrhea leads to an uptake of self-care strategies by women[23]. Complementary medicine usage (such as herbal medicines or traditional remedies) is often a significant component of self-care[23, 24]. Many women already use various forms of complementary medicine to manage their menstrual pain in addition to, or instead of, pharmaceutical pain relief, due to a lack of perceived effectiveness[19, 22] or a dislike of using analgesic medication[25].

The implications of normalising menstrual pain in conjunction with a lack of understanding of effective self-care strategies may lead to poor pain management. Given the link between pain intensity and negative outcomes, such as absenteeism, at school, university or work [26] ensuring that young women have access to effective self-care such as non-steroidal anti-inflammatories (NSAIDs) [27], the contraceptive pill [2] or effective non-pharmaceutical methods such as exercise[28] and heat [13] is vital. It is currently unclear what self-care strategies young women worldwide are using, if they are finding them effective, and what sources of information they are accessing to help make those decisions.

The aim of this systematic review and meta-analysis was to determine the prevalence and type(s) of self-care used for dysmenorrhea, the sources of information on menstruation used by young women under 25, and to explore any possible differences in both self-care and sources of information between low to middle income (LMIC) and high income (HIC) countries.

## Materials and methods

Preferred Reporting Items for Systematic Reviews and Meta-Analyses (S2 File—PRISMA) guidelines were adhered to throughout this review [29].

### Search strategy and selection criteria

**Fig 1:**
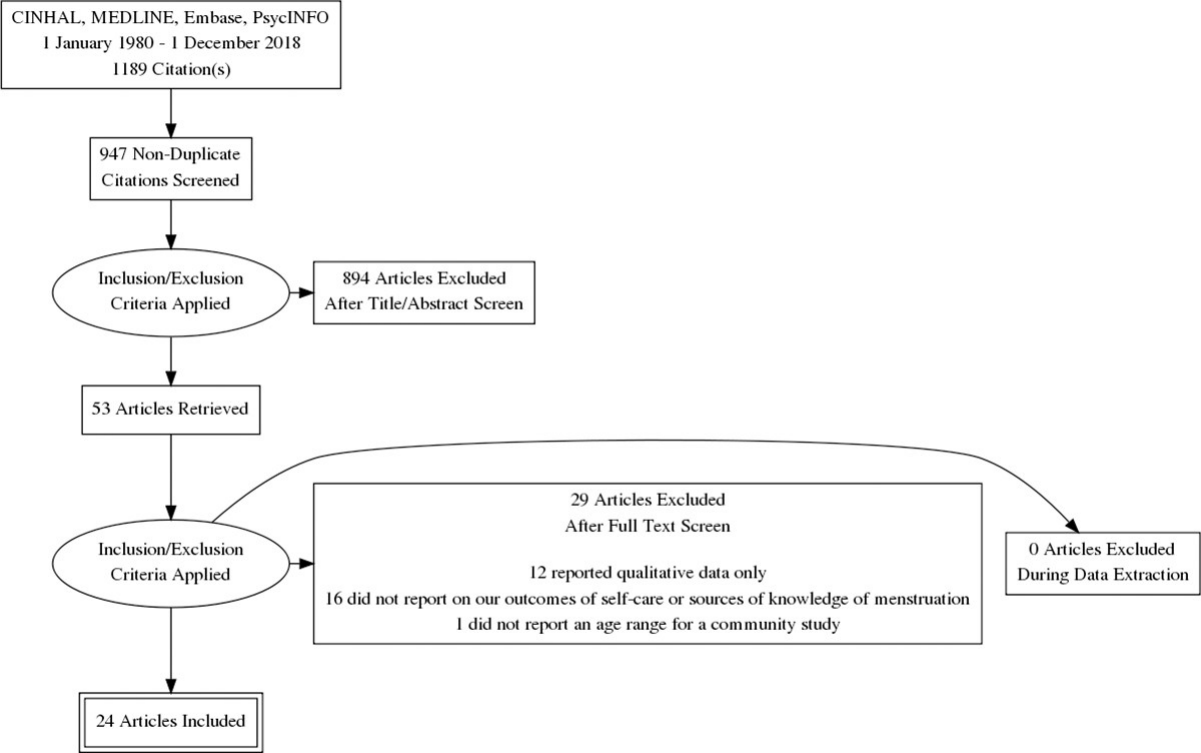
PRISMA flow diagram of the search and selection process.

A literature search was performed on Cumulative Index to Nursing and Allied Health Literature (CINAHL), Medline, Embase, and PsycINFO databases. All databases were searched from 1980 till 1 December 2018 using the following main keywords: ‘self-care,’ ‘management’, ‘symptoms’, ‘dysmennorhoea’ and ‘adolescen*’. The search and selection processes are outlined in Fig 1. The detailed search strategy is enclosed in S1 File. Only English language articles published in peer reviewed journals were included.

Observational studies (including cohort, cross-sectional, and case-control studies) were included. Studies were eligible if they specified that the participants were either at school or university (even if age was not reported) or were recruited from the broader community and included those aged ≤25 years. Eligible studies were those that included data on self-management strategies. Self-management could include either pharmacological (such as analgesic medication or the contraceptive pill) or non-pharmacological strategies (such as rest or herbal medicine). Studies which reported on secondary dysmenorrhea only were excluded as these would not represent the true prevalence rate or impact of dysmenorrhea. Studies which only reported qualitative data were not eligible for inclusion.

### Data extraction

Two authors (MA, KP) assessed eligibility, while three authors (MA, KP, MaD) extracted the data independently and any disagreements were resolved by discussion. Where data were missing or unclear, the study authors were contacted via email to request the data. Authors were contacted twice over a 6-week period, and if no response was received within that period, the data was marked as missing. A systematic tool for data extraction was developed to extract all relevant information from eligible studies.

Data were extracted on all of the following outcomes (if reported):

- Prevalence of dysmenorrhea
- Overall self-care usage (all categories)
- Overall analgesic usage (all categories)
- Usage of analgesics by category (Paracetamol, Ibuprofen and Other NSAIDs)
- Perceived effectiveness of analgesics (both overall and by category)
- Overall non-pharmacological self-care usage (all categories)
- Usage of the most common categories of non-pharmacological self-care (heat, rest, exercise, herbal medicines)
- Perceived effectiveness of non-pharmacological self-care (both overall and by category)
- Number and type of consultation(s) with health professionals (both overall and by category)
- Sources of information about menstruation (family, friends, medical professionals, teachers and the internet)

Study characteristics (location, demographics) were also extracted. Self-care usage, analgesic usage and non-pharmacological self-care were only included if overall figures were reported in studies (eg they were not assembled by adding together sub-categories (such as number using heat and number using exercise)) as these often-allowed multiple answers and would likely have given incorrect estimates. If effectiveness data was presented as a Likert scale (e.g. I think paracetamol helps my period pain) all positive scores (such as agree or most of the time) were considered as evidence of effectiveness while neutral and negative responses (such as no change or never) were considered as not showing evidence of effectiveness. Given the diversity of potential self-care approaches due to the cultural and geographic diversity of included studies, four common categories were chosen to allow a manageable analysis. This was based on a combination of our recent review of effective self-care in dysmenorrhea [13] as well as the authors expert opinion on common strategies. Rest included options such as laying down, having a nap and sleeping. Heat included hot baths, hot showers, heat packs or hot water bottles and herbal medicine included herbal teas, decoctions, pills or powders. Finally, if the type of medical or health professional was not specified, for example if women indicated they visited a clinic this was classified as ‘Visited Other Health Professional’ as there was insufficient data to determine the type of health professional seen.

### Quality assessment

Quality assessment of the reporting of the included studies was performed independently and in duplicate by all authors with any disagreements resolved by a third party. The quality of reporting in each study was assessed using a condensed version of the STROBE guidelines that have been used in previous reviews [30, 31]. The modified guidelines contain thirteen questions which covered reporting on; selection methods, validity and reliability of measures, methods to deal with bias, sample sizes and conflicts of interest. Each author rated bias items as yes (bias present) or no/unclear (bias may be present).

### Data synthesis and meta-analysis

A random-effects meta-analyses was conducted using Comprehensive Meta-Analysis software (Version 3). Data were pooled for each outcome where there were data from at least three independent studies.

A random effects model was used to account for expected heterogeneity between studies. Statistical heterogeneity between studies was quantified using Cochran’s Q and *I*^*2*^ statistic, both of which provide estimates of the degree of heterogeneity resulting from between-study variance, rather than by chance. Cochran's Q with p-value of <0.05 was classified as significant heterogeneity, and *I*^*2*^ of more than 75% was considered to indicate high level heterogeneity, *I*^*2*^ of 50%–75% as indicative of substantial heterogeneity, and an *I*^*2*^ of less than 40% as low heterogeneity.

Pre‐planned subgroup analyses were conducted to examine whether there was a difference in prevalence or impact in High income vs Low-Income, Lower-Middle-Income (LMIC) and Upper-Middle-Income (HIC) Economies (as classified by the World Bank[32]) due to differences in access to suitable medical and/or social support and between those at school and university/tertiary education to see if there were changes related to age or increased knowledge of menstruation. Sub-group analyses were conducted if there were a minimum of three studies for each sub-group.

## Results

Nine hundred and forty-seven studies were returned by our searches after duplicate studies were removed. Fifty-three full text articles were screened. Twenty-nine studies were excluded after screening; 12 due to reporting only qualitative data, 16 for not reporting on the use of self-care or sources of menstrual knowledge and one for not reporting an age range in a community-based study, Twenty-four studies including 12,536 young women were included in the analysis. All studies were cross-sectional quantitative studies. Nine studies (6,275 young women) were from HIC and 15 studies (6,261 young women) from LMIC.

Four studies were undertaken in Nigeria[33–36], three in Australia[15, 37, 38] and Iran [39–41], two in Hong Kong [8, 22] and Mexico [14, 42], and one each in the USA [7], Taiwan [43], Sweden [44], Sri Lanka [45], Saudi Arabia [46], Malaysia [47], India [48], Ghana [49], Ethiopia [50] and Brazil [51]. Study publication dates ranged from 1985 to 2018. Mean ages at the time of the survey ranged from 13 [51] to 23 years old [49], with a median age of 19. Twelve studies did not report the mean age of the participants, but still met the inclusion criteria; ten were undertaken in schools, one at both school and university and one community study [37] reported a valid age range (14–19) but not the mean age. Fourteen studies reported on young women at school, six reported on young women at university or higher education, three recruited from a community population; one using online recruitment, one a mixture of online and postal and one where it was unclear, and one recruited from both school and university. Table 1 summarises the included studies.

**Table 1:** Characteristics of included studies.

Study ID	Study Design	Country	Mean Age	Sample Size	Sample Population	Reports On
**Abidoye (2012)**	Cross-sectional quantitative	Nigeria	NR	180	School & University	• Overall Self-Care Usage • Analgesic/Pain Medication Use (Total) • Non-Pharma or Traditional Chinese Medicine Usage ○ Total ○ Rest ○ Heat
**Abraham (1985)**	Cross-sectional quantitative	Australia	NR Age range 15–19 years	1377	Community (unclear)	• Contraceptive Use • Sources of Advice: ○ Doctor ○ Friends ○ Teacher
**Adinma (2008)**	Cross-sectional quantitative	Nigeria	NR	550	School	• Analgesic/Pain Medication Use (Total) • Contraceptive Use • Non-Pharma or Traditional Chinese Medicine Usage ○ Total ○ Heat • Sources of Advice: ○ Doctor ○ Family ○ Friends ○ Teacher
**Alsaleem (2018)**	Cross-sectional quantitative	Saudi Arabia	19.1	197	University	• Prevalence of Dysmenorrhea • Analgesic/Pain Medication Use: ○ Total ○ Other NSAID • Non-Pharma or Traditional Chinese Medicine Usage (Total) • Sources of Advice: ○ Doctor ○ Family ○ Friends ○ Teacher
**Ameade (2018)**	Cross-sectional quantitative	Ghana	23	293	University	• Prevalence of Dysmenorrhea • Overall Self-Care Usage • Analgesic/Pain Medication Use ○ Total ○ Paracetamol ○ Ibuprofen ○ Other NSAID • Non-Pharma or Traditional Chinese Medicine Usage: ○ Total ○ Herbal Medicine • Effectiveness of Analgesics (Total) • Sources of Advice: ○ Got Medical Advice (Total) ○ Family ○ Friends
**Banikarim (2000)**	Cross-sectional quantitative	USA	NR	740	School	• Prevalence of Dysmenorrhea • Analgesic/Pain Medication Use (Total) • Non-Pharma or Traditional Chinese Medicine Usage ○ Rest ○ Exercise ○ Herbal Medicine ○ Heat • Sources of Advice: ○ Doctor ○ Nurse
**Chia (2013)**	Cross-sectional quantitative	Hong Kong	20.1	240	University	• Prevalence of Dysmenorrhea • Overall Self-Care Usage • Analgesic/Pain Medication Use ○ Total ○ Paracetamol ○ Other NSAID • Non-Pharma or Traditional Chinese Medicine Usage (Total) • Effectiveness of Analgesics: ○ Paracetamol ○ Other NSAID • Effectiveness of Traditional Chinese Medicine (Total) • Sources of Advice (Got Medical Advice Total)
**Chiou (2008)**	Cross-sectional quantitative	Taiwan	16.7	760	University	• Prevalence of Dysmenorrhea • Analgesic/Pain Medication Use (Total) • Non-Pharma or Traditional Chinese Medicine Usage ○ Rest ○ Exercise ○ Heat • Sources of Advice: ○ Doctor ○ Nurse ○ Family ○ Friends ○ Teacher
**Devi (2014)**	Cross-sectional quantitative	India	NR	100	School	• Analgesic/Pain Medication Use (Total) • Non-Pharma or Traditional Chinese Medicine Usage ○ Rest ○ Exercise • Sources of Advice: ○ Family ○ Friends
**Ghaderi (2017)**	Cross-sectional quantitative	Iran	21.3	197	University	• Prevalence of Dysmenorrhea • Analgesic/Pain Medication Use ○ Total ○ Other NSAID • Non-Pharma or Traditional Chinese Medicine Usage (Total) • Sources of Advice: ○ Family ○ Friends ○ Internet
**Hillen (1999)**	Cross-sectional quantitative	Australia	NR	388	School	• Prevalence of Dysmenorrhea • Analgesic/Pain Medication Use ○ Total ○ Other NSAID • Contraceptive Use • Non-Pharma or Traditional Chinese Medicine Usage ○ Total ○ Herbal Medicine • Effectiveness of Analgesics: ○ Paracetamol ○ Other NSAID • Sources of Advice: ○ Doctor • Nurse
**Moronkola (2006)**	Cross-sectional quantitative	Mexico	NR	120	School	• Analgesic/Pain Medication Use: ○ Total ○ Paracetamol ○ Other NSAID
**Ortiz (2009)**	Cross-sectional quantitative	Mexico	NR	1152	School	• Prevalence of Dysmenorrhea • Analgesic/Pain Medication Use: ○ Paracetamol ○ Other NSAID • Effectiveness of Analgesics (Total) • Sources of Advice (Doctor)
**Pitangui (2013)**	Cross-sectional quantitative	Brazil	13.65	218	School	• Prevalence of Dysmenorrhea • Analgesic/Pain Medication Use (Total) • Sources of Advice: ○ Got Medical Advice (Total) ○ Teacher
**Poureslami (2002)**	Cross-sectional quantitative	Iran	NR	250	School	• Prevalence of Dysmenorrhea • Analgesic/Pain Medication Use (Total) • Sources of Advice (Got Medical Advice Total)
**Rostami (2007)**	Cross-sectional quantitative	Iran	NR	660	School	• Prevalence of Dysmenorrhea • Analgesic/Pain Medication Use (Total) • Sources of Advice (Doctor)
**Saka (2018)**	Cross-sectional quantitative	Nigeria	15.2	400	School	• Prevalence of Dysmenorrhea • Overall Self-Care Usage • Analgesic/Pain Medication Use ○ Paracetamol ○ Other NSAID • Non-Pharma or Traditional Chinese Medicine Usage (Total) • Sources of Advice: ○ Got Medical Advice (Total) ○ Family ○ Friends ○ Teacher
**Söderman (2018)**	Cross-sectional quantitative	Sweden	16.2	1785	Community (postal invitation with online collection)	• Prevalence of Dysmenorrhea • Analgesic/Pain Medication Use ○ Total ○ Paracetamol ○ Ibuprofen • Contraceptive Use • Effectiveness of Analgesics (Total) • Sources of Advice: ○ Got Medical Advice (Total) ○ Doctor ○ Nurse
**Subasinghe (2016)**	Cross-sectional quantitative	Australia	21.5	247	Community (online)	• Prevalence of Dysmenorrhea • Analgesic/Pain Medication Use (Total) • Contraceptive Use • Non-Pharma or Traditional Chinese Medicine Usage (Total) • Sources of Advice: ○ Got Medical Advice (Total) ○ Family
**Sule (2007)**	Cross-sectional quantitative	Nigeria	NR	400	School	• Prevalence of Dysmenorrhea • Overall Self-Care Usage ○ Total ○ Paracetamol ○ Other NSAID • Sources of Advice: ○ Family ○ Friends
**Wijesiri (2013)**	Cross-sectional quantitative	Sri Lanka	NR	200	School	• Prevalence of Dysmenorrhea • Analgesic/Pain Medication Use ○ Total ○ Paracetamol ○ Ibuprofen ○ Other NSAID • Non-Pharma or Traditional Chinese Medicine Usage ○ Rest ○ Exercise ○ Heat • Sources of Advice (Family)
**Wong (2011)**	Cross-sectional quantitative	Malaysia	15.28	1295	School	• Prevalence of Dysmenorrhea • Analgesic/Pain Medication Use (Total) • Non-Pharma or Traditional Chinese Medicine Usage ○ Rest ○ Herbal Medicine ○ Heat • Sources of Advice: ○ Doctor ○ Family ○ Friends ○ Teacher
**Wong (2015)**	Cross-sectional quantitative	Hong Kong	15.69	531	School	• Analgesic/Pain Medication Use (Total) • Sources of Advice (Got Medical Advice Total)
**Yesuf (2018)**	Cross-sectional quantitative	Ethiopia	20.5	246	University	• Prevalence of Dysmenorrhea • Analgesic/Pain Medication Use ○ Total ○ Paracetamol ○ Ibuprofen ○ Other NSAID • Contraceptive Use • Non-Pharma or Traditional Chinese Medicine Usage: ○ Total ○ Rest • Sources of Advice (Doctor)

### STROBE assessment on reporting quality

The results of the STROBE assessment for the 24 studies are summarised in S1 Table. Twenty-three studies clearly reported their aim. Ten studies clearly reported their eligibility criteria, in addition one study reported the exclusion criteria only and one study reported very broad criteria. Thirteen studies used a form of random sampling. All 23 studies used questionnaires. Only five studies reported using validated measures, with three of these five studies lacking detail on how the measure was validated. Four studies reported a sample size calculation. Response rates ranged from 37% to 100%. Ten studies did not report response rates. Eight studies reported no conflict of interest, with one study reporting a potential conflict that was disclosed. The most common sources of bias were self-report bias, selection bias and responder bias, with recall bias also being present, but less common. Only two studies reported on methods used to minimise bias.

### Prevalence of dysmenorrhea

**Fig 2:**
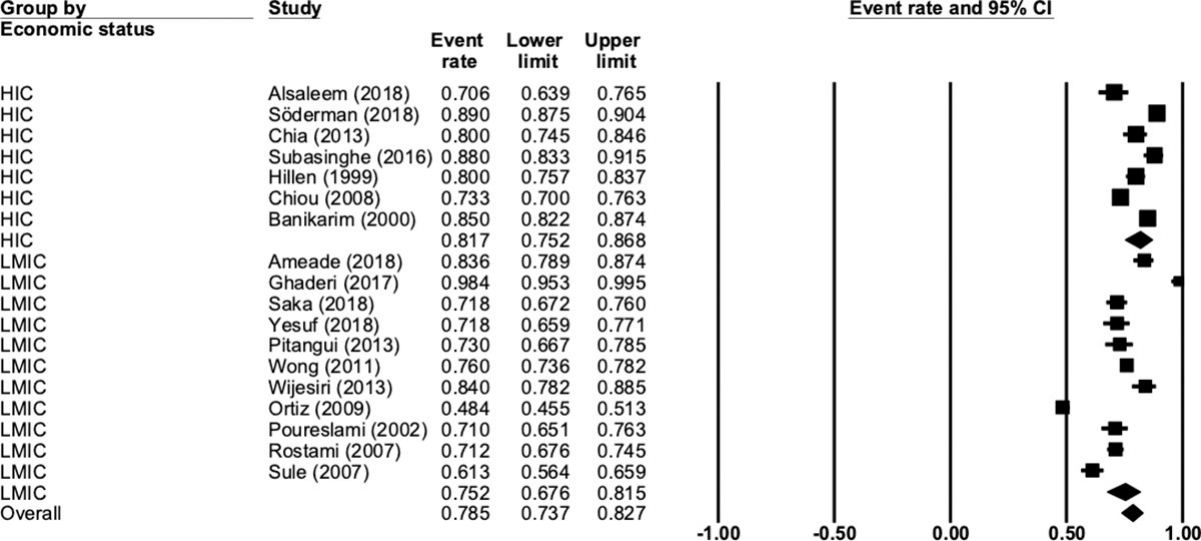
Prevalence of dysmenorrhea by economic status.

Overall prevalence rate for dysmenorrhea in the included studies was 78.5% (N = 18, n = 9668, 95% CI 73.7 to 82.7, Q = 718, P < 0.001, I^2^ = 97.6). When analysed by economic status, the prevalence of dysmenorrhea was higher in HIC countries at 81.7% (N = 7, n = 4357, 95% CI 75.2 to 86.8, Q = 124, P < 0.001, I^2^ = 95.1) than LMIC at 75.2% (N = 11, n = 5311, 95% CI 67.6 to 81.5, Q = 335, P < 0.001, I^2^ = 97.0), but this difference was not significant (p = 0.159). Fig 2 presents the complete data for this comparison.

### Self-care usage

Only five studies reported on the overall rate of self-care usage. Self-care was used by 55% of women in the included studies (N = 5, n = 1513, 95% CI 34.1 to 74.3, Q = 216, P < 0.001, I^2^ = 98.1). There were not enough studies reporting on this outcome to perform subgroup analysis.

### Pharmacological analgesia

**Fig 3:**
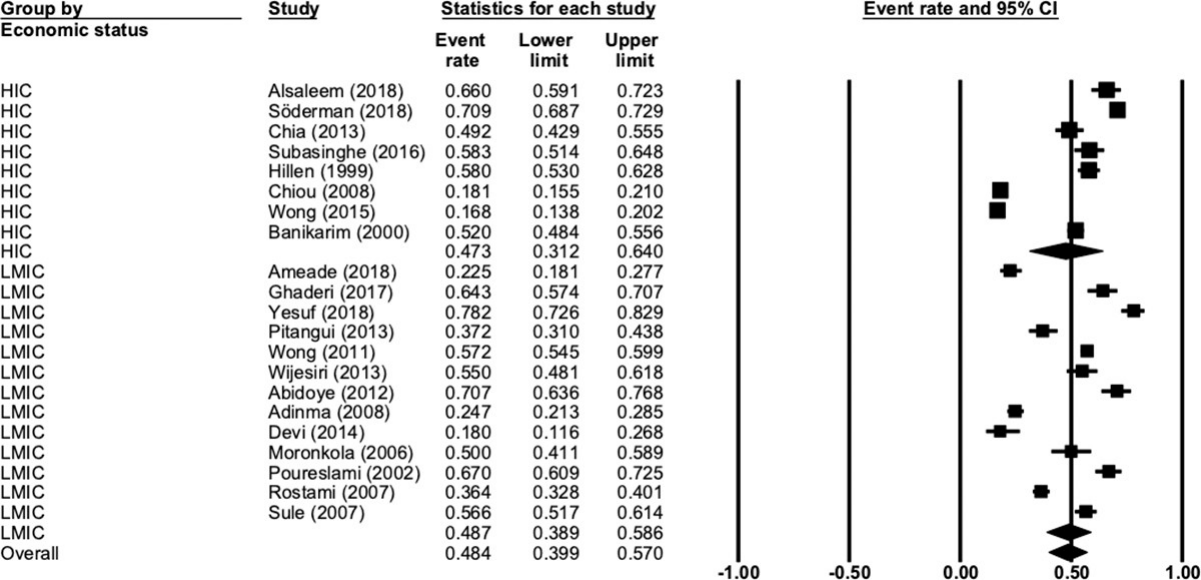
Pharmacological analgesia use.

Overall rates of pharmacological analgesia usage in the included studies was common, with just under half (48%) of women reporting using analgesics (N = 21, n = 9556, 95% CI 40.0 to 57.0, Q = 1246, P < 0.001, I^2^ = 98.3). Fig 3 presents the complete data for this comparison.

There was no difference (p = 0.89) between analgesic usage in LMIC (48.7%, N = 13, n = 4709, 95% CI 38.9 to 58.6, Q = 475, P < 0.001, I^2^ = 98.4) and HIC (47.3%, N = 8, n = 4847, 95% CI 31.2 to 64.0, Q = 757, P < 0.001, I^2^ = 99.1). There was also no difference (p = 0.653) in the rates of analgesic usage in young women at school (43.1%) and university (48.9%).

When looking at specific classes of analgesics, paracetamol was the most common (28.7%) (N = 9, n = 4836, 95% CI 19.6 to 39.9, Q = 396, P < 0.001, I^2^ = 97.9), followed by other NSAIDS such a naproxen sodium (23.3%) (N = 11, n = 3833, 95% CI 16.3 to 32.3, Q = 311, P < 0.001, I^2^ = 96.7) and finally Ibuprofen (17.1%) (N = 4, n = 2524, 95% CI 6.3 to 38.8, Q = 172, P < 0.001, I^2^ = 98.2).

Three studies reported on the effectiveness of analgesic medication overall, where 31.5% of women reported that analgesic usage reduced their pain (N = 3, n = 3230, 95% CI 22.9 to 41.6, Q = 57, P < 0.001, I^2^ = 96.5). Two studies looked at the self-reported effectiveness of paracetamol (57.8%, N = 2, n = 628, 95% CI 53.9 to 61.7, Q = 0.54, P = 0.46, I^2^ = 0) and other NSAIDS (97.3% N = 2, n = 628, 95% CI 24.9 to 1.0, Q = 11, P = 0.001, I^2^ = 91).

### Contraceptive use

Very few studies reported on contraceptive usage. In the six studies reporting on this, contraceptive usage was significantly higher (P<0.001) in HIC (22%, N = 4, n = 3797, 95% CI 11.0 to 39.3, Q = 268, P<0.001, I^2^ = 98) compared to LMIC (<1%, N = 2, n = 246, 95% CI 0.0 to 2.9, Q = 1.9, P = 0.165, I^2^ = 48).

### Traditional, complementary or non-pharmacological interventions

**Fig 4:**
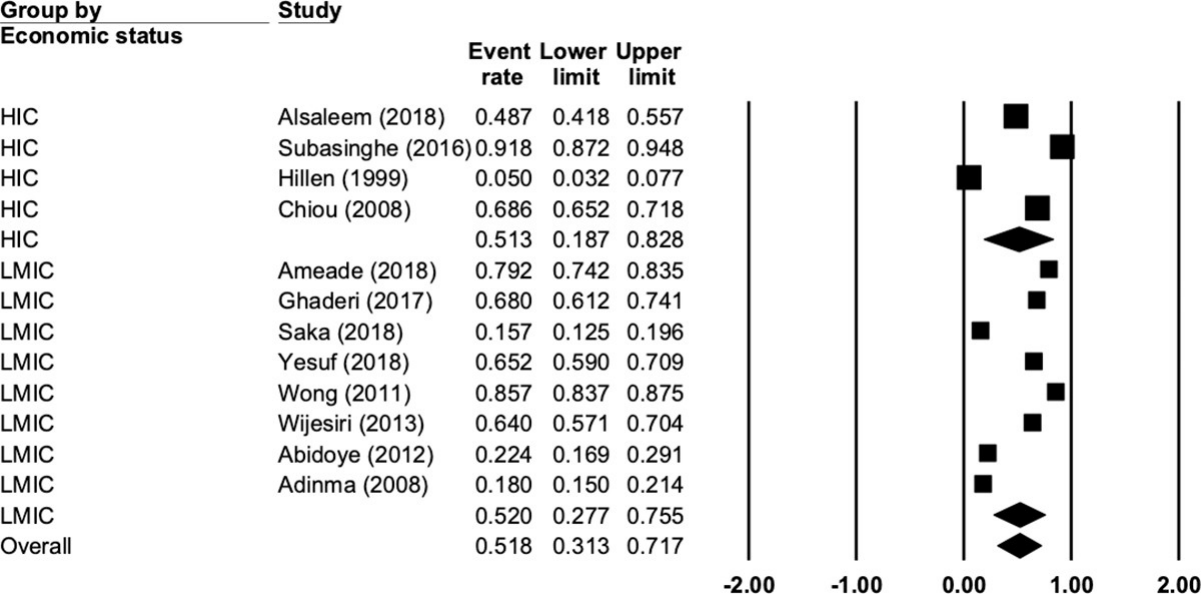
Complementary, traditional or non-pharmacological interventions.

Usage of complementary, traditional or non-pharmacological interventions to manage menstrual symptoms was common (51.8%, N = 12, n = 4913, 95% CI 31.3 to 71.7, Q = 1273, P < 0.001, I^2^ = 99.1). Fig 4 presents the complete data for this comparison. There was no difference (p = 0.974) between LMIC (52%, N = 8, n = 3361, 95% CI 27.7 to 75.5, Q = 970, P < 0.001, I^2^ = 99.2) and HIC (51%, N = 4, n = 1552, 95% CI 18.7 to 82.8, Q = 302, P < 0.001, I^2^ = 99.0).

**Fig 5:**
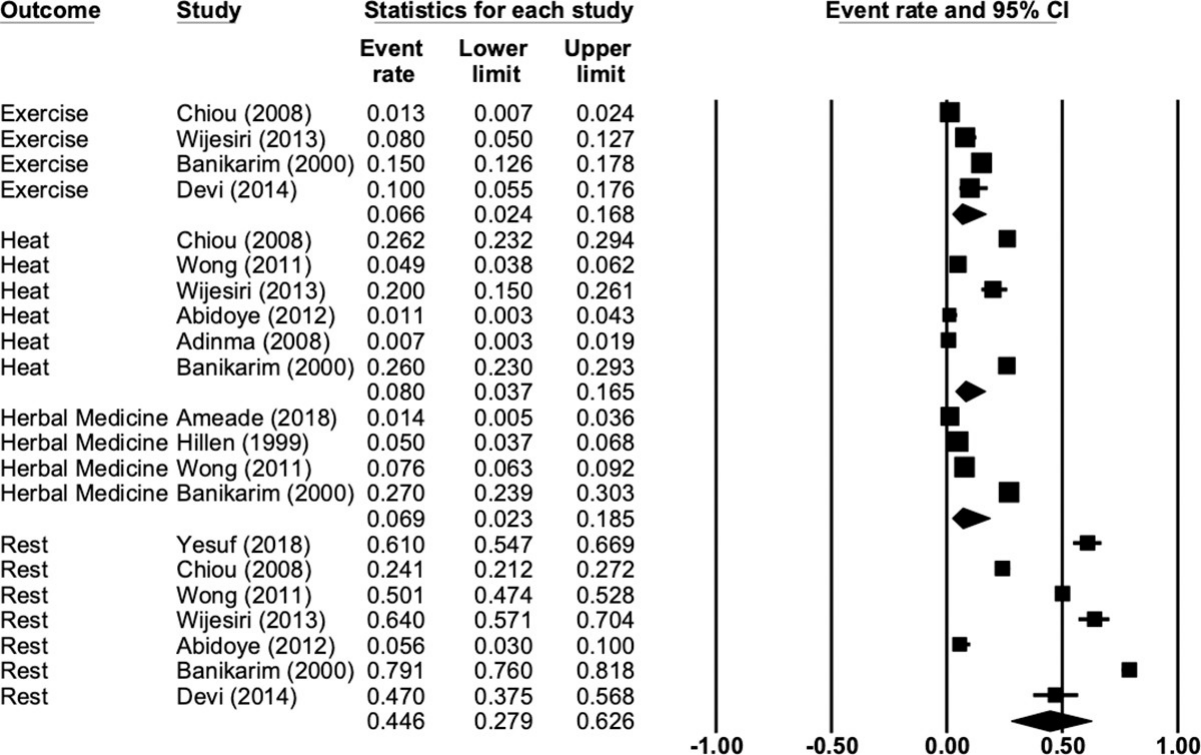
Traditional, complementary or non-pharmacological intervention comparison.

Rest was the most commonly used non-pharmacological intervention (44.6%) followed by heat (8%), herbal medicine or herbal teas (6.9%) and exercise (6.6%). Fig 5 presents the complete data for this comparison. There were not enough studies reporting on these outcomes to perform a subgroup analysis. There was only one study that reported on the effectiveness of these interventions, so no analysis was performed.

### Medical consultations

Only 11% of women in the included studies reported visiting a medical doctor to discuss their period pain (N = 11, n = 9150, 95% CI 8.4 to 15.2, Q = 252, P<0.001, I^2^ = 96). There was no difference in consultation rates between HIC (10.7%, N = 6, n = 5247, 95% CI 6.4 to 17.3, Q = 178, P<0.001, I^2^ = 97) and LMIC (11.8%, N = 5, n = 3903, 95% CI 8.1 to 16.8, Q = 70, P<0.001, I^2^ = 94). Four studies, all performed in HIC, reported that 22% of young women reported visiting a nurse, usually at school (N = 4, n = 3673, 95% CI 11.1 to 38.8, Q = 263, P<0.001, I^2^ = 98). In addition, 15.1% of women in the included studies reported seeking medical attention or advice (such as visiting a clinic) but the studies did not specify what kind of health professional they saw (N = 8, n = 3924, 95% CI 10.2 to 21.8, Q = 203, P<0.001, I^2^ = 96).

### Other sources of information

Outside of medical consultations the most common sources of information about menstrual symptoms was family (62.4%, N = 11, n = 4598, 95% CI 60.9 to 64.0, Q = 476, P<0.001, I^2^ = 97.9), friends (51.4%, N = 10, n = 5569, 95% CI 49.8 to 53.0, Q = 923, P<0.001, I^2^ = 99.0) and teachers/lecturers (22.8%, N = 7, n = 1377, 95% CI 21.5 to 24.1, Q = 306, P<0.001, I^2^ = 98.4). Only one study (n = 197) reported on the use of the internet (21.8%). There was no difference in rate of consulting these sources when comparing LMIC or HIC (all P>0.05). Young women at school (24.6%) were much more likely (P<0.001) to list teachers as a source of information on menstruation than those at university (3.6%).

## Discussion

The overall rates of dysmenorrhea (78%) in the included studies were similar to our previous meta-analysis on worldwide prevalence rates (71.1%)[1]. The usage of self-care or self-management strategies was relatively common with just over half of women reporting using self-care. Both non-pharmacological strategies and analgesic usage were similarly prevalent. Family and friends, along with teachers were common sources of information and play a significant role in providing information about menstruation.

Overall, analgesic usage did not provide sufficient relief to the majority of women taking it. Common NSAIDs, such as ibuprofen, naproxen and aspirin, are all superior to placebo for pain relief in primary dysmenorrhea, and there does not appear to be one particular type that is superior to others[52]. Paracetamol was the most common type of analgesic used by women in the included studies. This is a concern as paracetamol does not appear to be any more effective than a placebo[53] and is significantly less effective than NSAIDs [52] in reducing menstrual pain. This may explain why those women taking NSAIDs reported a much higher level of effectiveness compared to paracetamol. In the absence of prior medical consultation, adolescent self-medication has shown to lead to less frequent doses being taken resulting in sub-optimal pain relief[18, 54]. This is a serious concern as higher pain levels are strongly correlated with greater academic disruption[45].

The low usage of the oral contraceptive pill in the included studies is surprising, given that population surveys have shown an inverse relationship between contraceptive usage and self-reported period pain[55–57] and the combined oral contraceptive (COC) is commonly recommended as a second line of treatment for primary dysmenorrhea if NSAIDs have been unsuccessful [5, 58]. The reasons for the lower usage in LMIC may be influenced by cultural or religious factors, especially in young or unmarried women due to concerns that the COC promotes sexual promiscuity [12, 59]. Usage of COC in HIC was significantly higher, but still considerably lower than using analgesics, even though many women reported lack of effective pain relief with analgesics. This may at least in part stem from women’s concerns around long-term health consequences of the COC, or due to side effects [60, 61].

Rest was the most common non-pharmacological self-management strategy, with exercise, heat and herbal medicine being used by less than one in ten young women in the included studies. Exercise, especially low intensity exercise such as stretching or yoga, and heat are both effective self-management strategies for primary dysmenorrhea [13] and may be a useful alternative or adjunct to analgesic medications, especially in women who do not respond to analgesics.

Knowledge of dysmenorrhea from non-medical sources, such as family, friends and teachers were common. The normality of period pain may be reinforced by speaking to female family members (such as mothers or sisters) and peers as it is very likely that they will have had period pain[19, 62]. The concept of having to endure menstrual pain and symptoms as an integral part of female life is common amongst women with dysmenorrhea[6, 63–65], with even medical students not visiting their doctor when having period pain [66]. This is consistent with the concept that women “assemble” an idea of a normal period from their own experiences[67]. This normalisation of pain is likely to be a contributing factor to the usage of self-care rather than seeking medical intervention, leading to the low rates of visiting health professionals seen in the included studies.

There are a number of strengths in this systematic review and meta-analysis. We searched for articles across a range of databases and used dual data extraction via a pre-specified data extraction form to ensure rigorous data collection. Decisions on how to determine a country’s status used the four tier World Bank system[32] rather than the more simplified ‘developing’ or ‘developed’ bipartite classification. There are a number of limitations that must be acknowledged. First, we did not search in languages other than English, so there may be a number of non-English language papers that were not included. Second, there were significant inconsistences in reporting between surveys, especially with respect to the types of self-care used. Many surveys reported the use of regionally specific brand names for medications. While these were classified where possible, often information was not available in English. This may have resulted in under reporting the usage of specific medication types. Finally, the number of individual non-pharmacological self-care categories was limited to those which are commonly used in western countries and may not have represented the diversity of different self-care measures in different areas.

Since most young women do not seek medical advice, it would be prudent to ensure that the sources of advice they do consult; family, friends and teachers, as well as the young women themselves are suitably equipped. This could be achieved via well designed educational interventions; either delivered face to face, online or as a mixture of these. Teachers, in an Australian context particularly Health and Physical Education (HPE) teachers, can provide an effective means of providing all children and young people with the menstrual health literacy needed to manage menstruation and menstrual symptoms. With such a significant uptake of self-care options, teachers need to ensure they are informed on a range of effective complementary, traditional or non-pharmacological interventions to manage menstrual symptoms. Ideally this education on effective self-care would be given either prior to or approximating menarche, before the concept of period pain being ‘normal’ and needing to be ‘endured’ is firmly established in young women.

## Conclusions

Dysmenorrhea was common amongst young women, and the majority of self-care was undertaken without medical advice. Use of paracetamol, a less effective analgesic in treating period pain, was common compared to ibuprofen or other types of NSAIDs. Less than half of young women reported that they received satisfactory relief from analgesics in general. The use of the oral contraceptive pill was very low amongst young women in LMIC, likely due to cultural or religious beliefs around its use. Educational interventions which discuss the effective use of NSAIDs, and the incorporation of effective non-pharmacological management strategies, such as using exercise and heat, should be offered as these are likely to reduce the negative impact of dysmenorrhea on young women.

## Supporting information

S1 File Detailed search strategy. (PDF)

S2 File Selfcare PRISMA checklist. PRISMA Checklist.
(DOC)

S1 Table STROBE assessment. (PDF)
