# Supplementary material for: Self-care strategies and sources of knowledge on menstruation in 12,526 young women with dysmenorrhea: A systematic review and meta-analysis
Source: PLoS One. 2019 Jul 24;14(7):e0220103. doi: 10.1371/journal.pone.0220103 (PMC6655766; doi:10.1371/journal.pone.0220103)
Supplement: S1 File — (PDF) [file pone.0220103.s001.pdf]

Adolescen* OR child OR young OR paediatric OR pediatric* OR girls OR females OR school girls
AND
dysmenorrh* OR dysmenorrhoea OR menstrual cycle OR endometriosis OR menst* disorders OR menstrual pain OR painful menst* OR period pain
AND
Self-care OR symptoms OR practices OR management
