# Supplementary material for: Self-care strategies and sources of knowledge on menstruation in 12,526 young women with dysmenorrhea: A systematic review and meta-analysis
Source: PLoS One. 2019 Jul 24;14(7):e0220103. doi: 10.1371/journal.pone.0220103 (PMC6655766; doi:10.1371/journal.pone.0220103)
Supplement: S1 Table — (PDF) [file pone.0220103.s003.pdf]

	Aims	Selection methods			Was the measurement of variables appropriate?			Control of bias			Was the use of statistics appropriate?		Conflict of interest[1]
Study author and year	Aims clearly stated?	Eligibility criteria stated?	Source of participants described?	Selection method?	Validity of measures?	Reliability of measures?	Other method used?	Potential sources of bias?	Methods to deal with bias?	Response rate (%)?	Sample size?	Primary outcome stated a priori?	
Abidoye (2012)	Y	N/R	Y	Random	N/R	N/R	Questionnaire	Self-report, selection bias, responder bias	N/R	N/R	N/R	N/R	N/R

[1] N/R = Not reported, none stated = reported no conflict of interest, reported = reported potential conflict(s) of interest.

Abraham (1985)	Y	Y	Y	Random	N/R	N/R	Questionnaire	Self-report, selection bias	Y	N/R	N/R	N/R	None stated
Adinma (2008)	Y	N/R	Y	Random (stratified)	N/R	N/R	Questionnaire	Self-report	N/R	91%	N/R	N/R	N/R
Alsaleem (2018)	Y	Y	Y	Purposive	Y[2]	N/R	Questionnaire	Self-report, selection bias	N/R	N/R	N/R	N/R	None stated
Ameade (2018)	Y	Y	Y	Random	Y	N/R	Questionnaire	Self-report	N/R	70%	Y	N/R	None stated
Banikarim (2000)	Y	N/R	Y	Convenience	N/R	N/R	Questionnaire	Self-report, selection bias	N/R	N/R	N/R	N/R	N/R

[2] Authors state questionnaire has been validated but do not give sufficient information.

Chia (2013)	Y	Exclusion criteria	Y	Convenience	N/R	N/R	Questionnaire	Self-report, selection bias	N/R	93.75%	N/R	N/R	N/R
Chiou (2008)	Y	N/R	Y	Convenience	N/R	N/R	Questionnaire	Self-report, selection bias and recall issues	N/R	94.40%	N/R	N/R	N/R
Devi (2014)	Y	Y	Y	Random	N/R	N/R	Questionnaire	Self-report	N/R	N/R	N/R	N/R	N/R
Ghaderi 2017	Y	Y	Y	Random(cluster)	Y[3]	Y	Questionnaire	Self-report , possible selection bias (no reported	N/R	N/R	Y	Y	None stated

[3] Iranian version of previously validated questionnaire

								response rate)					
Hillen (1999)	Y	N/R	Y	Random	N/R	N/R	Questionnaire	Self-report, selection bias and recall issues	N/R	99.74%	N/R	N/R	N/R
Moronkola (2006)	Y	N/R	Y	Random	N/R	N/R	Questionnaire	Self-report	N/R	100%	N/R	N/R	N/R
Ortiz (2009)	Y	N/R	Y	Convenience	N/R	N/R	Questionnaire	Self-report, minimal demographic data, selection bias	N/R	N/R	N/R	N/R	None stated
Pitangui (2013)	Y	Y	Y	Unclear	N/R	N/R	Questionnaire	Self-report,	N/R	36.90%	Y	N/R	N/R

								selection bias due to low response rate					
Pour eslami (2013)	Y	N/R	Y	Random(cluster)	Y	Y	Questionnaire	Self-report, minimal demographic data	N/R	N/R	N/R	N/R	N/R
Rostami (2007)	N/R	N/R	Y	Random	N/R	N/R	Questionnaire	Self-report, minimal demographic data	N/R	89.40%	N/R	N/R	N/R
Saka (2018)	Y	Y (very broad)	Y	Random (multi-stage)	N/R	N/R	Questionnaire	Self-report	N/R	N/R	N/R	N/R	None stated
Söderman (2018)	Y	N/R	Y	N/R	N/R	N/R	Questionnaire	Self-report,	Y – Bias	45%	N/R	N/R	Reported

								and potential selection bias	analysis				
Subasinghe (2016)	Y	Y	Y	Convenience	N/R	N/R	Questionnaire	Self-report	N/R	69%	N/R	N/R	Reported
Sule (2007)	Y	N/R	Y	Convenience	N/R	N/R	Questionnaire	Self-report, minimal demographic data, selection bias	N/R	89.50%	N/R	N/R	N/R
Wijesiri (2013)	Y	Y	Y	Convenience	N/R	N/R	Questionnaire	Self-report, selection bias	N/R	100%	N/R	N/R	N/R

Wong (2011)	Y	Y	Y	Random	Unclear[4]	N/R	Questionnaire	Self-report	N/R	100%	N/R	N/R	None stated
Wong (2015)	Y	Y	Y	Convenience sample	N/R[5]	N/R	Questionnaire	Self-report, selection bias, responder bias	N/R	N/R	N/R	N/R	N/R
Yesuf (2018)	Y	N/R	Y	Random (stratified)	N/R	N/R	Questionnaire	Self-report	N/R	98%	Y	N/R	None stated

Supplementary Table 1. STROBE assessment

[4] Authors state questionnaire has been validated but do not give details.
[5] Validated scales (ADSCS and ESCAS) used in the overall survey, but the tool which provided the data used in this analysis was not validated.
